# Honey and Bee Products: Characterization, Bioactivities, and Authenticity

**DOI:** 10.3390/foods15040647

**Published:** 2026-02-11

**Authors:** Sandra M. Osés, Maria Teresa Sancho

**Affiliations:** Department of Biotechnology and Food Science, Universidad de Burgos (University of Burgos), Pza. Misael Bañuelos s/n, 09001 Burgos, Spain; mtsancho@ubu.es

Bee products, including honey, bee pollen, propolis, royal jelly, bee bread, and bee brood, are appreciated by consumers due to their sensory characteristics and health-promoting functions [1,2]. The fundamental step in establishing the therapeutic potential of honey and other apicultural products is to define their physicochemical, biological, and nutritional properties [3,4,5,6]. But also, to verify the authenticity of the products by different analytical techniques, as honey and other bee products can be adulterated [7,8,9]. In this context, the Special Issue, entitled “Honey and Bee Products: Characterization, Bioactivities and Authenticity”, contains nine research papers and one review. These articles highlight the importance of honey characterization to avoid adulteration. Fraudulent practices compromise the quality and authenticity of honey on a global scale, making studies on its potential biological properties and other bee products important for beekeepers and consumers.

Honey authenticity was assessed by different analytical techniques, each capable of discerning its botanical and geographical origins. Gürbüz and Kivrak demonstrated that an integrated approach—combining free amino acids and phenolic profiles (UPLC-ESI-MS/MS) with supervised chemometric methods—provided a successful and reliable model for determining the geographical origin of organic honeys from southeastern Türkiye. Barbarić et al. used GC-MS to reveal a unique volatile profile, distinguishing linden honeydew honey from other honeydew varieties. Their samples exhibited strong antimicrobial activities and good wound-healing capabilities, indicating that this honey possessed promising bioactive properties and dermatological potential. Dua et al. generated a database of isotopic signatures of Indian honey, where Δδ13CP-H values (>+1‰ equivalent to C4 sugar < −7%) were proposed as indicators of C3 adulteration. Honrado et al. used cytochrome C oxidase I (COI) metabarcoding to assess the honey bee source of European honeys. This technique showed high sensitivity. Honey’s entomological origins were accurately identified. It was C-lineage honey bees that mainly produced commercial honeys from northwestern Europe. Therefore, the entomological origin could be an indicator of adulteration for honeys with a protected designation of origin (PDO). Tasić et al. characterized Serbian honeys, revealing differences in their composition and quality. Irregular contents of sucrose, 5-hydroxymethylfurfural, acidity, and diastase activity could indicate adulteration.

Another important point of this Special Issue is the study of the biological properties of different bee products. Mokhtar et al. underlined the apicultural and ecological importance of *Vachellia tortilis* and *Ziziphus spina-christi* honeys in arid regions and reviewed the therapeutic value of these honeys from the United Arab Emirates. González-Montiel assessed the content of bioactive compounds and antioxidant activity of emulsions with propolis extracts during simulated in vitro digestion, showing a good emulsion stability that helped protect the bioactive compounds. Osés et al. evaluated the antimicrobial activity of honey from different botanical origins, concluding that all the honeys exhibited a high anti-*Staphylococcus aureus* activity and that catalase activity could be considered a possible marker of the antimicrobial activity of honeys against *S. aureus*. Tongchai et al. studied the food safety and antioxidant activity of worker honey bee (*Apis mellifera* L.) broods from Northern Thailand. The results showed that it is a safe, sustainable, and nutritious food with considerable antioxidants. Cavallero et al. studied Italian polyfloral bee pollen, showing good antioxidant, antiradical, antimicrobial, and anti-inflammatory activities that could alleviate the inflammatory responses of various chronic pulmonary conditions.

To conclude, this Special Issue highlights three pivotal themes: the importance of bee product characterization to avoid fraud, the necessity of using different analytical techniques to detect honey adulterations, and the relevance of studying the potential biological properties of bee products. All papers can help promote local beekeeping in each area and add economic value to bee products. This Special Issue features a collection of high-quality original research and reviews, providing readers with significant insights into the field.
